# Polyelectrolyte Multilayers: An Overview on Fabrication, Properties, and Biomedical and Environmental Applications

**DOI:** 10.3390/ma14154152

**Published:** 2021-07-26

**Authors:** Larisa-Maria Petrila, Florin Bucatariu, Marcela Mihai, Carmen Teodosiu

**Affiliations:** 1 “Petru Poni” Institute of Macromolecular Chemistry, 41A Grigore Ghica Voda Alley, 700487 Iasi, Romania; larisa.petrila@icmpp.ro (L.-M.P.); fbucatariu@icmpp.ro (F.B.); 2 Department of Environmental Engineering and Management, “Gheorghe Asachi” Technical University of Iasi, 73 D. Mangeron Street, 700050 Iasi, Romania

**Keywords:** layer-by-layer, polyelectrolyte, medical and environmental applications, emerging pollutants

## Abstract

Polyelectrolyte multilayers are versatile materials that are used in a large number of domains, including biomedical and environmental applications. The fabrication of polyelectrolyte multilayers using the layer-by-layer technique is one of the simplest methods to obtain composite functional materials. The properties of the final material can be easily tuned by changing the deposition conditions and the used building blocks. This review presents the main characteristics of polyelectrolyte multilayers, the fabrication methods currently used, and the factors influencing the layer-by-layer assembly of polyelectrolytes. The last section of this paper presents some of the most important applications of polyelectrolyte multilayers, with a special focus on biomedical and environmental applications.

## 1. Introduction

Since the introduction of the layer-by-layer (LbL) deposition model by Iler in the 1960s [1] and its development by Decher and Hong in the 1990s [2], the deposition of polyelectrolyte multilayers was considered an effective method for modifying surfaces, regardless of their shape, and found many uses in top fields of science: controlled-release of bioactive compounds, tissue engineering, biosensors, environmental remediation and pollutants retention, as well as the development of modern energy conversion and storage systems. Through their outstanding versatility, the surfaces functionalized with polyelectrolytes, especially with polyelectrolyte multilayers (PEMs), proved to offer suitable active colloidal supports for loading/release of bioactive compounds (drugs, proteins, enzymes, nucleic acids, aminoacids, etc.) or pollutants (dyes, pesticides, humic acids, heavy metal ions, etc.). Additionally, flat surfaces functionalized with polyelectrolyte multilayers can act as antimicrobial surfaces or, in case of using natural polyelectrolytes, as supports for tissue engineering. Due to the high densities of functional groups bound to a flexible polymeric chain, polyelectrolytes have a great potential in biomedical and environmental applications through the intimate interaction at the atomic level with various types of active (bio)compounds. Taking the advantages of polyelectrolyte characteristics, numerous types of organic architectures can be fabricated by covalent or non-covalent bonding (hydrogen bonding, π-π stacking, and metal-ligand coordination, ionic and hydrophobic interactions) between polyelectrolytes and different inorganic/organic partners. This review aims to highlight the importance of polyelectrolyte multilayer assembly in solving two main problems of humans (increased number of sick people and less clean water to satisfy the economic and societal needs) by creation of new formulations and advanced materials for drugs delivery and wastewater treatment, respectively [3,4]. The following sections present the general mechanism and methods used for LbL deposition of multilayers and the biomedical/environmental applications of PEMs.

## 2. Polyelectrolytes

**Figure 1:**
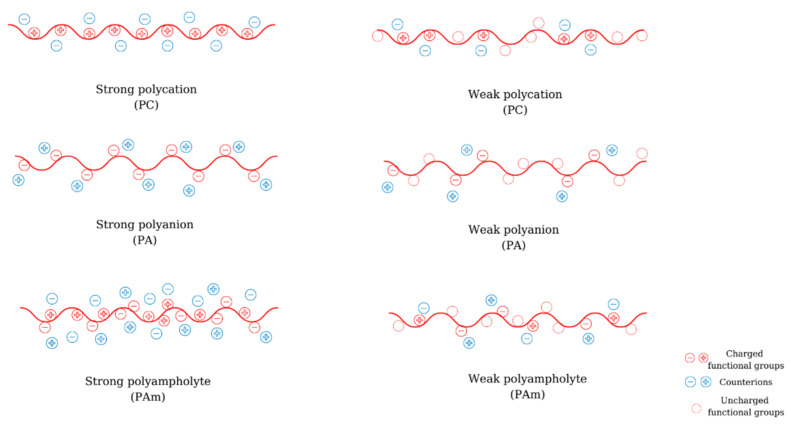
Classification of polyelectrolytes according to the nature of the functional groups and the ionic charge.

Polyelectrolytes (PEs) are macromolecular compounds that possess minimum of 10–15% functional groups that are dissociated or dissociate in solution. Depending on their origin, polyelectrolytes can be natural (e.g., chitosan (CS), alginic acid, hyaluronic acid (HA), etc.), semi-synthetic (e.g., xanthan, modified CS), or synthetic (poly(ethyleneimine) (PEI), poly(acrylic acid) (PAA), poly(methacrylic acid) (PMA), etc.) Depending on the nature of the ionic charge, polyelectrolytes can be classified into polyanions (PA) (polyacids, have negative charges), polycations (PC) (polybases, have positive charges), polyampholytes (PAm) (amphoteric, have both positive and negative charges) (Figure 1). According to their dissociation behavior, PEs can be weak (their degree of ionization is depending on pH and their dissociation constant varies between 2 and 10) or strong (they dissociate completely in solution and their ionization degree is not affected by the pH).

To assess the degree of dissociation of a PE, potentiometric titrations can be performed, to obtain information about the acidity constant and the number of functional groups. Polyacids can be titrated with bases and polybases can be titrated with acids. The degree of dissociation of weak PEs is dependent on the number of functional groups in the macromolecular chain and can be calculated using the formula for the neutralization degree (Equation (1)):

*α* = *α*′ + *C_H_^+^*/*C_PE_* (1)

where, *C_H_^+^* is the molar concentration of *H*^+^ ions and *C_PE_* is the molar mass of the PE.

The apparent acidity constant (*pK_app_*_(*PE*)_) can be calculated using a modified version of the Henderson-Hasselbalch Equation (2):

*pK_app_*_(*PE*)_ = *pH*-*m*·*log*(*α*′/(*α*′−1)) (2)

where *m* can be found from the experimentally performed titrations, as a slope of the curve *pH* = *f*(*log*(*α*′/(*α*′−1)) [5].

PEs can also be classified according to their composition (homopolymers and copolymers) or molecular architecture (linear, branched, or cross-linked). Ionizable functional groups of the PEs can be located on the main chain (“integral” PE) or on the side chain (“pendant” PE).

## 3. Fabrication Methods for Polyelectrolyte Multilayers

**Figure 2:**
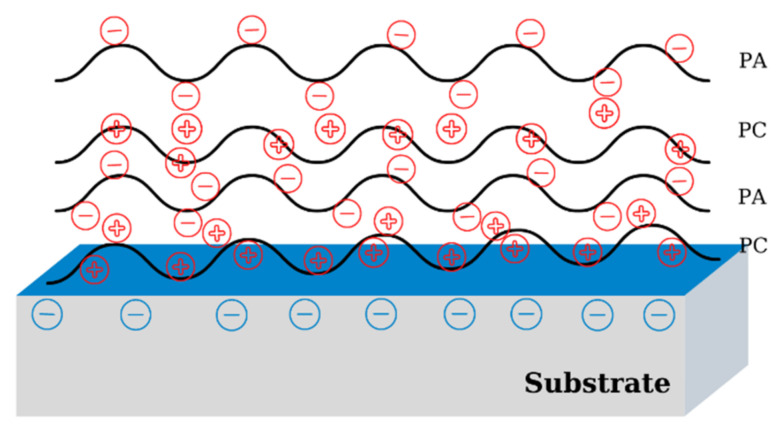
Schematic representation of a polyelectrolyte multilayer.

Polyelectrolyte multilayers are thin organic films obtained by self-assembly of PEs with other charged/uncharged (macro)molecules using a LbL method. The physical and chemical architecture of PEMs can be determined from the nanoscopic level to the macroscopic level. Both weak and strong PEs can participate in the formation of multilayers together with other low molecular or macromolecular compounds (charged or uncharged), the properties of the obtained materials being dependent on the nature and characteristics of the partners involved in the deposition process. The substrates commonly used in LbL deposition can have different forms, with different chemical surface compositions. Sometimes, the charged surfaces based, mostly, on inorganic oxides are only activated by washing with acidic-basic solutions. By oxidative acidic media, the organic contaminants of solid surface are destroyed and therefore the active sites for sorption are available. Instead, the acidic surfaces will be activated in basic media by creation of new negative charges, for example by Si–O–Si hydrolysis. In the case of inert surfaces, some aggressive physico-chemical treatments must be carried out, such as plasma treatment or chemical grafting [3,4]. The fabrication of multilayers is achieved mainly by successive adsorption of PEs on an activated substrate (Figure 2). In addition to the electrostatic interactions, covalent bonds, hydrogen bonds, van der Waals forces, or hydrophobic interactions can also contribute to the formation of multilayers [6,7].

The first layer is formed by the adsorption of a PE on the surface of a support possessing opposite charges. Subsequently, PE layers can be adsorbed, with the formation of a nanostructured film with the structure Substrate/(Polycation/Polyanion)_n_ or Substrate/(Polyanion/Polycation)_n_, as a function of the ionic charge of the substrate. The substrate used as a template in the LbL deposition can remain as a part of the final material or can be removed through chemical or physical treatment, allowing the fabrication of a free-standing multilayered material. Another peculiarity of multilayers is that, depending on the substrate used, they can be obtained as flat or three-dimensional surfaces. In the latter case, by dissolving the substrate, capsule-type structures can be obtained, which can incorporate various types of active compounds [7].

The increase in multilayer thickness depends on the number of layers deposited and can be either linear or non-linear, the deposition process being influenced by several parameters, such as ionic strength, pH, temperature, macromolecular structure, concentration, and charge density of PEs [8]. Although the mechanisms leading to the formation of a multilayer with linear or exponential growth are not fully understood, several studies address this issue, the growth mechanism being attributed to the dissociation of ionizable functional groups, secondary interactions or diffusion of PEs through the formed layer [9,10,11,12].

**Figure 3:**
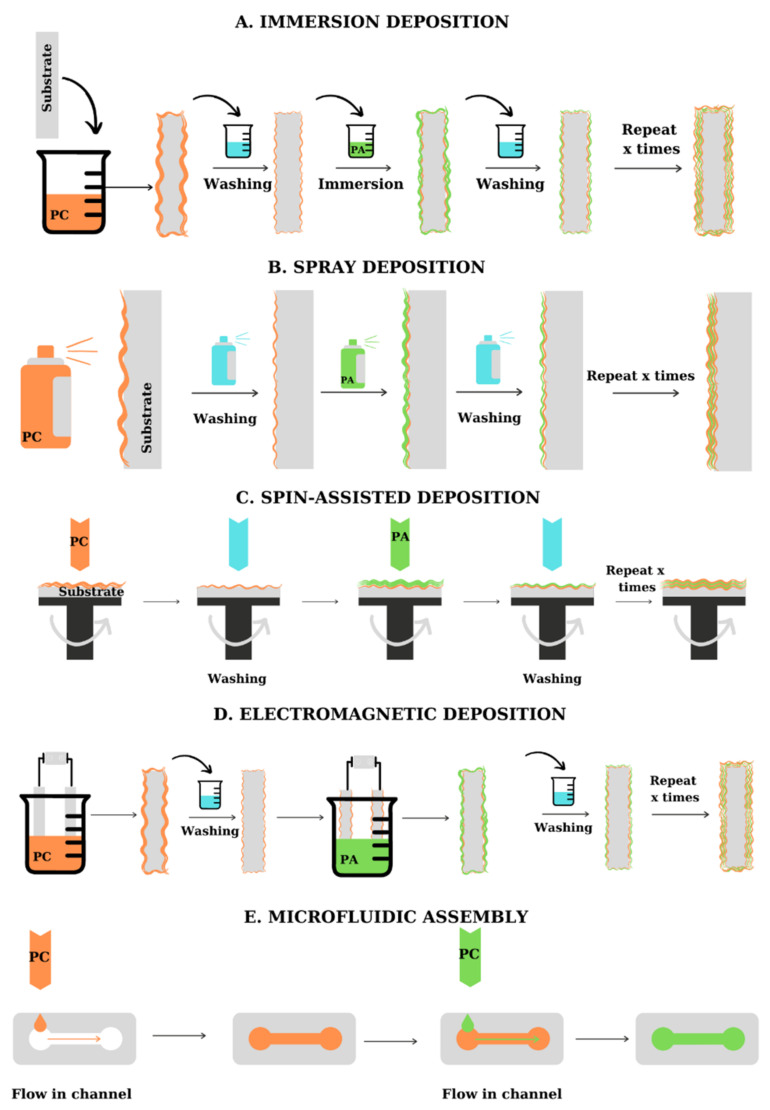
Schematic representation of the main LbL methods used for the fabrication of PEMs. (**A**) Conventional immersion deposition of PEs on flat substrates, (**B**) Spray deposition of PEs on flat substrates, (**C**) Spin-assisted deposition of PEs on flat substrates, (**D**) Electromagnetic deposition of PEs on flat substrates, (**E**) Microfluidic assembly of PEs on microfluidic materials.

From the practical point of view, multilayers can be fabricated by several methods [7]: dipping, spray deposition, centrifugal deposition, electrodeposition, and microfluidic deposition, as summarized in Figure 3. The choice of the deposition method depends on the materials to be assembled, the time required for the deposition, and the shape of the substrate. PEs can be successfully deposited on various substrates, including flat and tridimensional materials, porous and microfluidic materials, and even biological molecules. Figure 3 presents the main methods used for the fabrication of PEMs on planar substrates (A–D) and microfluidic materials (E).

Dipping deposition of polyelectrolyte multilayers involves the use of three components: a substrate with ionic charges on the surface, a polycation, and a polyanion (Figure 3A).

The substrate is immersed in the solution (usually aqueous) of the PE having an ionic charge of opposite sign, the electrostatic interactions leading to the formation of the first layer; after the adsorption of the first layer, the substrate is washed to remove the excess of PE. Afterward, the substrate is immersed in the solution of the second PE, washed, and subjected to a new deposition step. The washing between the PE deposition steps is particularly important to avoid the cross-contamination with polyelectrolyte complexes formed during the assembly [4]. Dipping deposition is a lengthy process; magnetic stirring, the addition of dimethylformamide into the PE solutions (in order to eliminate the washing step [13] or manual stirring can be used to reduce the deposition time, by favoring the fast interaction between the PE and the already adsorbed layer [14]. The thickness and roughness of multilayered films fabricated by dipping are higher than that of similar PEMs obtained by other deposition methods. For example, Elosua et al. [15] assembled poly(sodium phosphate) and poly(allylamine hydrochloride) (PAH) by dipping and spraying. The thickness of the materials obtained by dipping deposition of the PEs increased with the number of bilayers while, for the materials obtained by spraying, the thickness measured was lower.

Spray-assisted deposition of polyelectrolytes is a quicker fabrication method and allows the fabrication of multilayers with a predetermined thickness, based on the change in deposition conditions, and can be applied to large substrates (Figure 3B) [16]. To facilitate the deposition of the PE layer, the spraying deposition is performed on supports placed perpendicular to the direction of application of the PE solution, which ensures the removal of excess deposited material due to gravitational drainage. The method was firstly proposed by Schlenoff et al. [17] which alternately sprayed poly(styrene sulfonate) (PSS) and poly(diallyldimethylammonium chloride) (PDADMAC) on a support, obtaining LBL films with high uniformity and good quality. The thickness and roughness of the films obtained by spraying are lower than in the case of dipping deposition, but still present an increasing mechanism dependent on the number of bilayers. By testing the deposition of PEs on a silica template using horizontal spray deposition, vertical spray deposition, and dipping deposition, Alongi et al. [18] observed that the best results in terms of film homogeneity were obtained using horizontal spray deposition. Dierendonck et al. [19] highlighted the recent developments in the spray-assisted deposition of PEs. As presented by the team, the PEMs obtained by spray deposition present a more regular surface. Hsu et al. [20] used the spray-assisted deposition of PEs for the encapsulation of lysozyme. Testing several variations for the assembly, they optimized the deposition process, obtaining films with good enzyme encapsulation efficiency. The loss in raw material was also reduced. To avoid the deposition of an inhomogeneous film, the spraying method can be coupled with spinning [8,14].

Spin-coating deposition of polyelectrolyte multilayers involves the rotation of the support at a constant velocity simultaneously with the deposition of PE solutions (Figure 3C). The formation of a uniform polymeric layer is the result of electrostatic and centrifugal forces and air sheer [14,21]. The method has the advantage of allowing the formation of uniform multilayers and requires a lower quantity of material. In the case of PEMs formed by spin-assisted deposition, the interpenetration of adjacent layers is suppressed which leads to obtaining materials with a smoother surface than in the case of PEMs fabricated by the dipping of PEs [22]. Although it comes with certain advantages, the method is limited to the deposition on flat supports [16], the fabrication of structures with the desired characteristics being also influenced by the spinning rate and the concentration of PE solutions [8]. Cho et al. [23] demonstrated that air sheer plays a key role in the surface appearance of the assembled film. They assembled PAH and PSS multilayers using spin-assembly and dipping methods. The atomic force microscopy graphs registered for the multilayers obtained by the two methods showed that the roughness of the assembled PEMs is dependent on the deposition technique. For the film obtained by dipping, the surface roughness was 8.1 Å, while for the film obtained by spinning deposition, the surface roughness was 3.1 Å, which suggests that in the case of spin deposition, the PEs are absorbed in a flatter conformation. Similar results were obtained by Kiel et al. [24] which assembled PSS, PAH, and gold nanoparticles using the spin-assisted approach. The low roughness of the PEMs produced was confirmed by micrographs while other characterization techniques were used to analyze the characteristics of the obtained multilayers.

Electrodeposition of polyelectrolyte multilayers uses two electrodes immersed into a deposition cell, followed by the application of a potential difference (Figure 3D). The electrode coated with the deposition substrate and the reference electrode are immersed in a PE solution with opposite charge to the substrate and subjected to the action of an electric current that induces the formation of the polyelectrolyte film. After the deposition of the first layer, the substrate is washed and immersed in the solution of the second PE. This method is very fast and allows the control of the thickness of the multilayer formed, by changing the applied voltage, contact time, or the concentration of the solutions of used PEs [8,16]. However, the method is limited to the deposition of a small number of layers [8]. Zhang et al. [25] studied the effect of the electric field applied on the LbL deposition of PDADMAC and PSS. The results obtained suggest that the applied voltage influences not only the growth mechanism but also the thickness and the morphology of the film. Electrodeposition was successfully applied for the fabrication of enzyme/PE multilayer by Shi et al. [26]. The PEM was prepared by electric field-induced LbL assembly of different enzymes and PDADMAC onto an indium-tin oxide covered glass electrode surface. By testing the performances of the enzyme-covered electrode, the authors observed that the electrochemical signal is enhanced by the increase in the number of bilayers deposited on the electrode. More recently, Wang et al. [27] used the electrodeposition method to cover a titanium substrate with CS and alginate (ALG). By testing the cytotoxicity of the assembled PEMs, the team demonstrated the biocompatibility of these materials. A similar deposition method is based on the use of the magnetic properties of certain compounds. The substrate is introduced into the solution of compounds with magnetic properties and subjected to the action of a magnetic field that leads to the organization of molecules with the formation of a uniform film [8].

Microfluidic assembly of polyelectrolyte multilayers is used for the deposition of layers of polyelectrolytes on the walls and in the capillaries/channels of some microfluidic systems (Figure 3E). The alternating introduction of PEs solutions into the microfluidic assembly is usually carried out under vacuum or pressure. The method is faster than dipping deposition and allows the formation of polymeric films in materials with special forms for which other deposition methods cannot be applied [16]. From the beginning of 2000s, several authors [28,29,30] reported the LbL assembly of PEs on microfluidic materials, highlighting the fast deposition and the uniformity of the multilayers obtained. Madaboosi et al. [30] used the microfluidic assembly method for the fabrication of poly-L-lysine (PLL)/HA films, observing that the PEs supply rate is one of the key parameters influencing the deposition. Wang et al. [31] proposed a model of electrodeposition and microfluidic coupling for LbL deposition of CS and ALG.

The methods presented above refer to the deposition of PEs on macroscopic surfaces. However, since many of the applications of PEMs are related to the deposition of PEs on substrates such as colloidal particles, liposomes, or biological molecules, it is necessary to develop methods for the deposition of charged compounds onto such templates. The general method involved in the fabrication of PEMs on microscopic or nanoscopic templates is the dipping deposition and its characteristics and control methods are similar to those presented above. The method is presented in more detail in Section 6.1.

## 4. Factors Controlling the Fabrication of Multilayers

The fabrication of multilayers by LbL deposition is a versatile method, which allows efficient control of the deposition parameters. By changing the deposition conditions (pH, temperature, ionic strength) or the used PEs (molar mass, charge density, linear or branched structure) and the solvent, materials with different structures and properties can be obtained [14,32,33]. Many studies in the early 2000s dealt with the influence of pH, temperature, ionic strength, and other factors which have an influence on the LbL assembly of PEs, demonstrating the feasibility of tuning the properties of the PEMs.

The ionic strength is one of the most important parameters controlling the formation of PEMs as demonstrated by the studies focused on this topic [34,35,36]. The charge screening effect of the counterions, determined by the modification of the ionic strength, leads to structural changes in the conformation of the PEs involved in the formation of multilayers, which turn from a “stretched” to a “coiled” conformation. Thus, their ability to interact with each other leading to the formation of multilayers is affected, and changes in the stability and physical characteristics of multilayers may occur [37]. Some authors [38,39] showed that there is an upper limit for the ionic strength used in multilayer construction, after which the influence of this parameter is negligible, the main force driving the LbL assembly shifting from electrostatic interaction to other types of forces. The value of this parameter depends on the pair of PEs involved in the formation of the multilayer. The swelling and shrinkage properties of multilayers are also influenced by the ionic strength. In solution, PEs charges are compensated by counterions. When PEs form LbL, their charges are compensated by the charges of the opposite PE. When salt is added to the solution, the LbL film swells and some of the charges on the PE chains are compensated by the ions in the solution. Kharlampieva et al. [40] studied the influence of ionic strength on PEMs obtained by spin-assisted deposition. The multilayers obtained from salt-free solutions exhibited a well-defined structure, while in presence of salt ions, the layers were interdiffused. Guzmán et al. [41] also observed that the ionic strength influenced the interdiffusion of the PEs within the multilayer, leading to changes in the growth regime. Similar results were observed by Tang and Besseling [42] while studying the LbL assembly of PDADMAC and PSS at different NaCl concentrations. Hernandez-Montelongo et al. [43] studied the influence of pH and ionic strength on the LbL deposition of HA and CS, observing that both parameters influenced the topography, roughness, and thickness of the multilayers obtained. The thickness of the films increased with the number of bilayers for all the pH/ionic strength variation tested, the PEMs assembled at 0.1 M NaCl being thicker than the ones assembled at 0 M NaCl.

The solution’s pH may also control the formation and properties of multilayers formed with weak PEs [44,45,46]. In the case of weak PEs, such as PAA or PAH, small variations in pH cause modifications in the charge density (the ionization degree of the functional groups), leading to the modifications in the PEs conformation, in the thickness of the adsorbed polyelectrolytic films, in the swelling capacity of the multilayer, and its subsequent interactions with other compounds [47]. As reported by Guzman et al. [48], the pH mainly influenced the deposition of weak PEs, such as PAA, which diffused better in the neighboring layers at low values of pH, leading to the adsorption of a thicker layer. At high values of pH, the diffusion of PAA into the opposite-charged layers is inhibited. Barrantes et al. [49] studied the LbL deposition of PLL and heparin on silica and gold substrates as a function of pH. The PEMs constructed using acidic solution exhibited a flatter surface on the silica substrate due to the strong electrostatic attraction between the positively charged PLL and the negatively charged SiO_2_.

The solvent influences the solubility and ionization degree of the PEs [50]. Most of the PEMs are built using aqueous solutions. The use of water as a solvent will determine a high degree of ionization of the functional groups which will electrostatically interact with each other contributing to the formation of multilayers. The lack of toxicity and the economic advantages of water are also to be considered. The use of different solvents can lead to conformational changes in the PEs and, as a result, to changes in the structure and stability of the polyelectrolyte film formed. Additionally, the use of organic solvents will decrease the influence of electrostatic interactions in the formation of PE films as a result of the decrease in the dissociation degree of the PEs which also leads to their depletion from solution and the deposition on the already formed multilayers [8]. For the same reason, the use of water/organic solvent mixtures increases the thickness of the formed films for strong PEs as well as for weak PEs. Studying the LbL assembly of PSS and PAH on ethanol solutions, Poptoshev et al. [50] observed that a higher concentration of ethanol in the solution leads to the formation of a thicker film as a result of the reduction in the solvation effect. Chen et al. [51] also studied the effect of solvent in the LbL assembly of poly(8-(4-carboxy-phenoxy)-octyl acrylate) and poly(3-(4-pyridyl)-propyl acrylate) in solvent mixtures of tetrahydrofuran and ethanol. Testing different compositions for the solvent mixture, the authors observed that the solvent influenced the conformation of the polymer which leads to different assembly behaviors.

Temperature influences the solubility and the degree of ionization of PEs. Its effect on the thickness of the deposited PEs layers, in the swelling capacity, and in the stability of the multilayer is studied by several authors [52,53,54]. Studying the effect of temperature on the buildup of poly(diallyldimethylammonium) (PDADMAC)/PSS and PAH/PSS multilayers, Salomaki et al. [53] noticed that the growth regime is influenced by temperature, being, in general, exponential at higher temperatures. The growth regime switches to a linear one when the diffusion of PEs through the already deposited layers is slow while the increase in temperature leads to the destruction of electrostatic interactions between PEs and to conformational rearrangements. Vikulina et al. [54] studied the influence of temperature in the LbL assembly of PLL and HA multilayers, observing that the increase of temperature increased the thickness of the deposited layers.

In addition to these parameters, the formation of multilayers by LbL deposition is influenced by the intrinsic properties of the PEs. The charge density, the molar mass, the conformation, and the concentration of the PE solutions control the growth mechanism and properties of the multilayer. Charge density plays a key role in the LbL assembly since the main factor leading to multilayer formation is the interaction between oppositely charged functional groups. The influence of charge density on the assembly of PEs was assessed by many researchers [55,56,57,58]. A low charge density generally leads to the formation of thin PE films, while a too high charge density will induce the delamination of the film. As reported by Choi and Rubner [58], the assembly of PEMs using fully charged PEs lead to the formation of a thin film, while a lower degree of ionization leads to the formation of thicker films. Moreover, when investigating the self-assembly possibilities of strong PEs, several authors showed that the formation of multilayers is possible only above a minimum value of the charge density. Below this, the interaction between PE and the superficial layer is weak, leading to desorption of the last layer adsorbed and formation of inter-polyelectrolyte complexes dispersions [55,59,60,61].

The molar mass of polyelectrolytes is also a factor of interest [62,63,64]. Jang et al. [64] have shown that the molar mass of PEs influences the stability of the obtained multilayer, thus eliminating the need for further stabilization steps. A low molecular mass will increase the risk of PE desorption at the contact with the oppositely charged PE, while a higher molecular mass will enhance the deposition of multilayers and will increase its stability. Additionally, it is generally accepted that PEMs consisting of high molecular mass PEs are more stable than PEMs based on low molecular mass PEs [65,66,67]. Studying the LbL deposition of PLL and HA, Shen et al. [68] also observed that the molecular weight of HA influences the deposition of PEMs. The use of high-molecular mass HA leads to faster film growth. In a 2020 study, Towle et al. [65] followed the influence of molecular mass on three pairs of PAA/PLL (1.8 k/15–30 k, 100 k/120 k, and 250 k/275 k), highlighting that the mass and rigidity of the obtained PEMs increased with the increase of the PEs molecular mass. The concentration of the PE solutions must be high enough to ensure the deposition of stable layers and to allow the charge inversion in systems for which the LbL assembly is the result of electrostatic interactions. The minimum concentration required for the formation of multilayers depends on the characteristics of PEs [8]. In general, the use of higher concentrations of PEs leads to the formation of thicker multilayers because of the chain rearrangements of the PEs to ensure binding sites on the surface, as observed by Guzmán et al. [69] who studied the LbL assembly of PSS and a triblock copolymer containing poly (2-(N,N-dimethylamino)ethyl methacrylate)) and poly(ε-caprolactone). Similar results were observed in a subsequent study [70] where the authors tested the LbL assembly of PDADMAC/PSS and PAH/PSS multilayers at different concentrations and ionic strengths.

## 5. The Main Expected Properties of Multilayers

**Figure 4:**
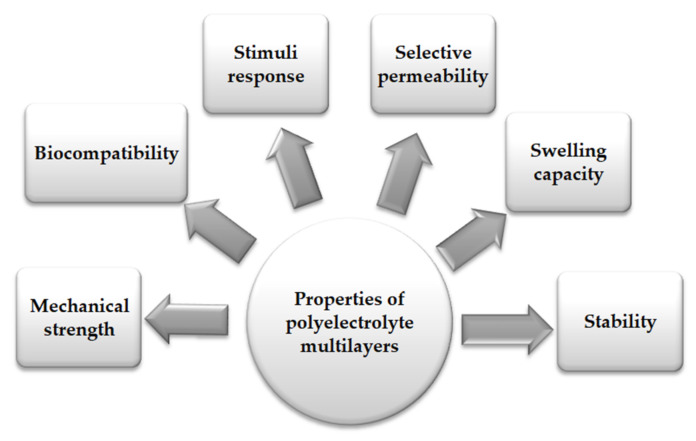
The main usually expected properties of polyelectrolyte multilayers.

The functional properties of multilayers are closely related to the chemical structure of the PEs used, but also to the applied deposition method, which can influence the swelling capacity, roughness, or mechanical properties of the multilayer [7]. The main factors that influence the properties of PEM-like materials are the building blocks used in the assembly of multilayers which lead to the fabrication of materials with different characteristics. The main usually expected properties of multilayers are presented in Figure 4.

The swelling capacity of multilayers is an important property in applications such as controlled-release systems and water purification, influencing the diffusion of small molecules through the multilayer. The water embedded in the multilayers influences the molecular interactions between the PEs [8,71]. The swelling behavior of PEMs is influenced by the ionic strength but also by the relative humidity in the environment, as shown by Dodoo et al. [72]. They studied the swelling behavior of PSS/PDADMAC multilayers prepared at different NaF, NaCl, and NaBr concentrations. The water content in the multilayers increased with the increase in ionic strength and ion size. In a subsequent study, the same team reported the influence of the deposition conditions on the water content for PSS/PDADMAC multilayers prepared at two ionic strength values; for the multilayers prepared at 0.1 M NaCl, the water content decreased with the increase in the number of bilayers for the PEMs with a small number of deposited layers while for materials with more bilayers, the water content was higher. For the multilayers prepared at 0.5 M NaCl, the water content increased with the number of double-layers deposited [73]. The influence of counterions on the swelling behavior of PSS/PDADMA was assessed by Salomäki and Kankare [74] which observed that the presence of fluoride ions did not affect the swelling of the PEMs, while in presence of bromide ions, the PEMs exhibited different degrees of swelling. Another factor influencing the swelling behavior of PEMs is the nature of the last deposited layer because of the different architecture the PEMs will adopt. As a result of a lower number of electrostatic interactions, the last deposited layer can embed higher quantities of water. It was also observed that the use of hydrophobic PEs as building blocks for the fabrication of PEMs will lead to thicker multilayers because their lower solubility will result in the depletion of the material from the solution [8].

The mechanical properties of multilayers depend on the macromolecular structure of the PEs used in the formation of multilayers, as well as on the self-assembly conditions and amount of water retained in the system. Depending on the water content, multilayers may have a gel-like behavior or may be rigid, their applications being closely related to the material flexibility [8]. Additionally, the mechanical properties of organic-inorganic composites allow their use in many fields due to their specific structure; the inorganic part is generally rigid, while the polymeric multilayer has a flexible structure [8,16]. As presented by some authors [75,76], multilayers composed of inorganic compounds such as montmorillonite, metal oxides, or ceramics and PEs exhibit very good mechanical properties while PEMs composed only by PEs have a more flexible structure. It was also observed that for the multilayers in which the PEs are adsorbed in a coiled conformation, the structure of the obtained material is more flexible, allowing better response to external stimuli [8]. Additionally, several studies [77,78,79] highlighted the influence of ionic strength on the mechanical behavior of PEMs. It was observed that as the ionic strength increases, the PEMs shift from elastic behavior to fluid-like behavior.

The stability of the multilayers is particularly important in various practical applications. The stability depends mostly on the ionic strength and solution pH and on the acido-basic characteristics of the complementary functional groups. The stability can be enhanced by cross-linking, which involves the formation of chemical bonds between the deposited PE layers and can be achieved by several methods (chemical, thermal, thermo-chemical, physical), between the deposition stages or after the fabrication of the multilayer [80,81,82,83]. The cross-linking by chemical treatment with various reactive agents leads to the formation of covalent bonds within the functional groups of the oppositely charged polyelectrolytes involved in the formation of the multilayers. Of these methods, the most used are thermal [84,85], and chemical (glutaraldehyde (GA) [86,87,88,89,90], pyromellitic dianhydride [91], epichlorohydrin [92,93,94], and genipine [95,96]) cross-linking. The cross-linking can be done in a step-by-step procedure, which implies the treatment of the multilayer with the cross-linking agent after each cycle of PE deposition. The step-by-step cross-linking has been applied experimentally in a series of studies, demonstrating the method’s feasibility [86,97,98]. Cross-linking can also be conducted after the fabrication of the multilayer, this procedure having the advantage of being faster than the step-by-step cross-linking. By cross-linking the PEs chains the functional properties of the obtained PEMs can be enhanced. The cross-linking degree influences the loading/release properties of multilayers. Bucatariu et al. [88] obtained poly (vinyl amine) (PVA)/PAA and PEI/PAA multilayers which were cross-linked with different ratios of GA. The use of the obtained composites in sorption and desorption of anionic dyes demonstrated that the amount of compound loaded and released from the multilayers was dependent on the degree of cross-linking, better results being obtained for the multilayers (PEI/PAA)_4.5_ cross-linked with 1% GA (the highest amount of cross-linker used). Similar results were obtained by Xu et al. [99] which fabricated PEMs based on PAA, PVA, and CS and thermally cross-linked. The multilayers obtained were subsequently used for the loading and release of gentamicin, demonstrating a good loading/release capacity for the cross-linked materials.

Many multilayers are made of *biocompatible* PEs. This class mainly includes multilayers formed using natural PEs (e.g., CS, heparin, ALG, HA, proteins, etc.). There are also several synthetic polyelectrolytes with good biological compatibility (e.g., PAA, poly(ethylene glycol) (PEG), poly(vinylpyrrolidone) (PVP)). Biocompatible PEMs mimic natural structures that are recognizable by the human organism and their degradation products can be metabolized, which allows their use in food, medical, and cosmetic applications. For example, Dai et al. [98] assessed the biocompatibility of a CS/collagen PEM deposited on a PCL/N6 mat. The obtained results demonstrated that the deposition of CS highly improves the biocompatibility of the material suggesting it can be used in biomedical applications. Amorim et al. [100] also reported the construction of biocompatible LbLs based on HA and PLL. The obtained PEMs were cross-linked with N-(3-dimethylaminopropyl)-N’-ethylcarbodiimide hydrochloride and N-hydroxysuccinimide and their interaction with CD44 protein was assessed. The results obtained suggested that the cross-linked PEMs were more stable and they specifically bind to CD44 without the disruption of the multilayered film. Arias et al. [101] also reported the adhesion and proliferation of cells on PSS and poly(diallyldimethylammonium) PEMs.

Another important property of PEMs is their selective permeability. This characteristic is essential in ensuring the exchange of matter with the external environment, making possible applications in the pharmacological and medical field, but also in the field of pollution management and environmental protection. The porosity of the obtained material is influenced by the nature of PEs and can be modified by the contribution of other parameters (pH, ionic strength, temperature, mechanical deformation, ultrasounds). At the same time, the rigidity of the obtained structure can lead to a sharp decrease in the permeability of multilayers [8]. Antipov et al. [102] studied the permeability of PEs-based capsules fabricated by LbL assembly of PSS and PAH. The results obtained suggest that the permeability of the capsules can be easily controlled by changing the pH and ionic strength which supports their use for the incorporation and release of active compounds. The permeability of the multilayers is also important for PEM-based membranes. Studying the filtration performance of a PDADMAC/PSS nanofiltration (NF) membrane, De Grooth et al. [103] observed that the deposition conditions influence the permeability of the membrane obtained. Similar observations were made by other researchers, as presented in Section 6.2.

PEMS can respond to induced stimuli. The stimuli-responsiveness of these materials is an important property for applications such as drug-delivery or biosensing. Since the beginning of 2000s, several authors have proposed different stimuli-responsive PEMs [102,104,105,106]. One of the first examples of stimuli-responsive multilayers is presented by Antipov et al. [102] which fabricated PAH and PSS based capsules which were subsequently used for the incorporation of FITC-labeled dextran and albumin, at different pH values. The obtained results demonstrated that the PEMs based capsules have a tunable permeability for the tested compounds, based on the pH changes. PEMs can also be responsive to enzyme action, which can degrade the PEMs leading to the release of active compounds retained into the multilayers [107]. The development of bacteria on specific PEMs could also trigger a specific response. For example, Francesko et al. [108] constructed HA and aminocellulose nanospheres LbL which exhibited antimicrobial activity against *Pseudomonas aeruginosa*. Similarly, Albright et al. [109] observed that the release of antibiotics from LbL capsules can be triggered by the development of microorganisms and Craig et al. [110] studied the controlled release of vancomycin or polyhexamethylene biguanide from a HA/PLL film under the presence of enzymes originated from *Pseudomonas aeruginosa* and *Staphylococcus aureus.*

## 6. Biomedical and Environmental Applications of Polyelectrolyte Multilayers

Multilayers can be sensitive to external stimuli (pH, temperature, radiation, ultrasound, etc.). These factors influence not only the characteristics of the formed multilayer but also the subsequent behavior and interaction with low-molecular weight compounds. The use of multilayers in various applications is based on the advantages that LbL deposition methods have, including facile deposition conditions, the possibility to use a large number of materials, and the possibility to obtain systems with different characteristics depending on the deposition conditions [64]. Possible applications of these PEMs focus on many directions, including pollutant retention, biosensors, energy storage and conversion, nanomedicine, and tissue engineering.

### 6.1. Biomedical Applications

**Figure 5:**
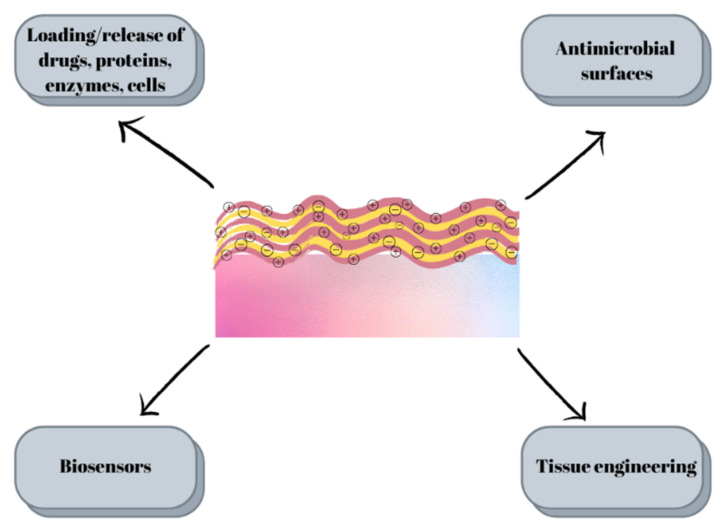
The main biomedical applications of PEMs.

The LbL deposition of PEs can be used for the fabrication of composite materials for biomedical applications, which implies the use of templates for the deposition of PEs including colloidal particles, inorganic materials, and even biological compounds. A large number of materials can be used as building blocks to obtain multilayers for biomedical applications. These include natural and synthetic polyelectrolytes, proteins, nucleic acids, viruses, lipids, dyes, nanoparticles, multivalent ions, dendrimers, surfactants, nanotubes, and nanowires that can be assembled into multilayers. In the early 2000s, Caruso et al. studied the possibilities of self-assembling proteins and nucleic acids into multilayers [111,112], obtaining materials with special purposes. Some authors have also fabricated multilayers using PEs and low molecular weight compounds [113], metal ions [114], and nanoparticles [115]. The general deposition mechanism is similar to the deposition onto macroscopic surfaces. The deposition of multilayers follows the same principle: the substrate is dispersed alternately in the opposite-charged PE solution, the PE is adsorbed on the surface, and the excess of PE is separated by centrifugation or sedimentation. The use of low-density particles such as liposomes or nanomaterials as substrates for the LbL deposition of PE can be difficult since centrifugation or sedimentation can’t be used for the separation of the multilayered materials from the deposition solution [8]. The most important biomedical applications of the multilayers are the incorporation and release of active substances (drugs, enzymes, proteins, liposomes) [116,117], tissue engineering [118,119], the fabrication of films with antimicrobial properties [120,121,122], and biosensing [123,124]. Figure 5 highlights the main biomedical applications of PEMs, which are briefly described in this section.

#### 6.1.1. Loading and Release of Active Compounds

**Figure 6:**
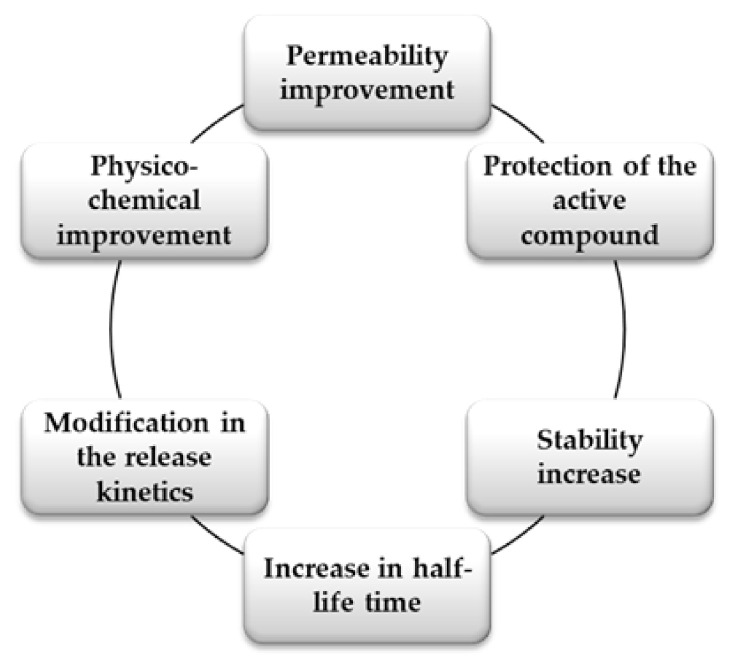
The advantages of multilayer encapsulation of active compounds.

Multilayers are widely used for the incorporation of active compounds, having several advantages that make them ideal for this type of application, as presented in Figure 6:

The incorporation of active compounds into multilayers ensures the improvement of stability and physico-chemical properties and protection of the active compound, allowing the release of the active substance in a controlled manner. As shown by Andreeva [71], the encapsulation of active compounds can ensure protection against different stress factors, such as exposure to light, humidity, or the human organism’s internal conditions by creating a barrier between the active compound and the environment. The possibility of modifying the release kinetics of active compounds by their encapsulation in multilayers is also important for controlled-release applications, based on the use of stimuli-responsive PEs as building-blocks for the deposition of multilayers [125]. The swelling behavior of the PEMs can also influence the release of the active compound.

The incorporation of active compounds into multilayers can be achieved either by their retention inside multilayer capsules or by their adsorption within the multilayer, with the formation of (polyelectrolyte/active compound, AC)_n_ systems. The multilayered systems for pharmaceuticals include porous nanoparticles, liposomes, thin films, microcapsules, and nanocapsules [126]. Table 1 presents some examples of active compounds incorporated into multilayers for facilitating their absorption.

**Figure 7:**
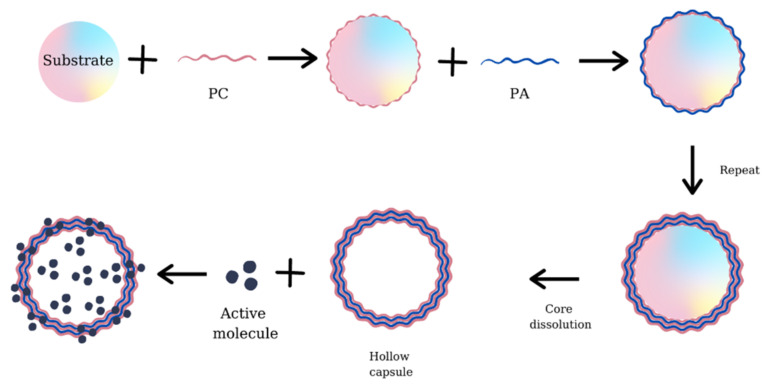
The mechanism of obtaining LbL hollow capsules and their use in the encapsulation of active compounds.

The fabrication of hollow capsules is obtained by LbL deposition of PEs on a charged support. The substrate can be represented by silica [128], polystyrene [106], nanoparticles [137], melamine formamide [138], or other charged materials. To obtain the empty capsules, the support is dissolved by chemical, physical, or enzymatic treatment. The properties of multilayered capsules depend on the deposition conditions, temperature, ionic strength, and the number of layers deposited. The advantage of these capsules is that their permeability can be influenced by external stimuli, leading to changes in the release mechanism of active compounds. Figure 7 presents the mechanism of obtaining LbL hollow capsules and their use in active compounds sorption/encapsulation.

Kumar et al. [128] presented an example for the encapsulation of an active compound in multilayered capsules. Using the LbL deposition method, the authors obtained polyvinylpyrrolidone (PVP)/PMA multilayered films deposited on a silica substrate. The substrate was dissolved with HF and $NH_{4}F$ leading to the formation of hollow capsules. The capsules were used to incorporate rifampicin, an antibiotic with antituberculosis action. The amount of antibiotic retained in the capsules was determined based on spectrophotometric measurements, noting that as the temperature increases, the amount of active substance encapsulated increases (4.5 mg/L at 4 °C and 14 mg/L at 40 °C).

Another method for incorporating active compounds is represented by their retention in the multilayer as a result of electrostatic interactions. The incorporation of the active compound can be achieved either after the fabrication of the multilayer or between the PE deposition steps. The mandatory condition for this incorporation method is that the drug has ionic charges, usually opposite to that of the surface PE layer, thus allowing electrostatic interactions between the components.

Bucatariu et al. [97] incorporated diclofenac sodium salt and indomethacin in a multilayer consisting of PEI and cross-linked with pyromellitic dianhydride and 3,3′, 4,4′-benzophenonetetracarboxylic dianhydride. The authors observed that the mass of active compound retained in the multilayer was influenced by the number of layers of polycation deposited on the support and by the amount of cross-linker used. For the multilayer consisting of PEI and diclofenac, the amount of active substance retained was about 4 mg/g composite, while in the case of indomethacin, the amount of active adsorbed compound was about 5 mg/g composite.

Another example is presented by Pilicheva et al. in an article published in 2020 [131]. The authors incorporated benzydamine, a therapeutic agent with anti-inflammatory, analgesic, antipyretic, and antimicrobial action, into an 8-layered system obtained from CS, casein (CAS) and poly (lactic acid), and cross-linked with GA, calcium chloride, and sodium tripolyphosphate (NaTPP). The authors were able to retain in the multilayer significant amounts of active compound, highlighting the effect of cross-linking on the active compound retention. The best results, in terms of drug retention, were obtained for the multilayers cross-linked with GA/calcium chloride (94.9 µg/cm^2^) and GA/sodium tripolyphosphate (217.4 µg/cm^2^), respectively, compared to the retention of only 13.8 µg/cm^2^, in the case of non-cross-linked multilayers [131].

Multilayers can also be used to incorporate biological molecules, in order to increase their stability under physiological conditions or to facilitate their transport into the organism. Enzymes, viral vectors, proteins, and genetic material can be included in this category. A large number of enzymes have been immobilized in PEMs, including catalase [139], cholesterol oxidase [140], glucose oxidase [141,142], lipase [143,144], lysozyme [86,145], pepsin [86,144], and peroxidase [146,147]. For example, Bucatariu et al. studied the immobilization of pepsin and lysozyme in a multilayer consisting of PEI cross-linked with BTCDA and GA. The amount of enzyme immobilized in the composite was correlated with the isoelectric point (iep) of each enzyme, the pepsin (iep = 1), being immobilized in a higher amount compared to lysozyme (iep = 10) [86].

Other possible applications of multilayers include the incorporation of genetic material for the treatment of heart disease, of DNA or RNA for vaccine delivery systems [148,149], of antigens [150], and growth factors [151]. Oliveira et al. [116] reviewed the most important techniques used in LbL coating of cells, presenting some up-to-date examples of biological materials incorporated in PEMs. Multilayer-type systems that incorporate genetic material offer a wide range of possibilities, as demonstrated in a study by Mandapalli et al. [151] that incorporated TGF-β and EGF growth factors and RNA sequences in a multilayer based on CS and ALG, and used it for wound healing. The fabrication of cardiac stents on a metal structure, covered with PEMs and incorporating DNA, has also been studied [152,153].

The release of active compounds from the systems in which they are incorporated can be achieved as target-delivery at specific sites (cancer cells, liver cells, small intestine etc.), under the action of external stimuli that cause the degradation of the protective coating. External stimuli such as pH, ionic strength, radiation, and ultrasound can trigger the release of the active compound in the surrounding environment either at a specific target cell or at a specific time span. The main mechanisms of controlled-release of active compounds from multilayers were presented by Wohl et al. [125].

After the incorporation of rifampicin into the multilayer, Kumar et al. [128] studied the release mechanism under physiological conditions (in phosphate-buffered saline, at 37 °C and pH = 7.4), as well as the antimicrobial activity of the capsules. The complete release of rifampicin from the capsules was achieved in about 20 min, being strongly influenced by pH. Regarding the antimicrobial activity of the capsules containing the antibiotic, studies performed on *Mycobacterium smegmatis* strain showed that the drug maintains its therapeutic activity even encapsulated, the ability to inhibit the growth of the microorganism being similar to that observed at the use of non-encapsulated rifampicin.

The release of benzidamine from the multilayer CS-CAS proposed by Pilicheva et al. [131] on the tests performed in phosphate buffered saline ensured conditions similar to those in the human body (pH = 6.8, 37 ± 0.5 °C). Following in vitro tests, it was observed that the release of the active compound had a very high rate in the case of non-cross-linked multilayers (over 90% benzidamide in 30 min), while in the case of cross-linked multilayers, the release rate of the active substance was slower, reaching equilibrium after about two hours.

Additionally, by determining the adhesion capacity of multilayers on a biological system simulating the characteristics of the oral cavity, it was shown that the multilayered films have a satisfactory mucoadhesion (absorption of 26.7–35.4% mucin, due to the interaction between the positive functional groups of CS and the negative ones of mucin), demonstrating that these multilayers are suitable for releasing the active substance in the mouth.

A special category of therapeutic agents that are suitable for immobilization in multilayers is represented by chemotherapeutic drugs. Characterized by high cytotoxicity, these drugs require inclusion in a protective matrix and are used as target-release drugs, so that the active compounds target the tumor cells. An example of the retention of chemotherapeutic agents in multilayers is presented in a study by Wang et al. [133] that constructed a multilayer based on CS and dextran sulfate for embedding paclitaxel and fluorouracil. The active compounds were encapsulated with an efficacy of 66.3% (paclitaxel) and 75.2% (fluorouracil) in the multilayer. In vitro controlled-release studies have shown that the release of the active substance is dependent on pH variations, which indicates the efficiency of using these systems to attack tumor cells, the difference between the pH of normal cells and that of tumor cells being a factor that favors the controlled-release. Cytotoxicity analysis showed that nanoparticles containing the active substances have cytotoxic action on cancer cells [133].

#### 6.1.2. Tissue Engineering Applications

PEMs are also used in the field of tissue engineering due to their ability to interact with biological molecules such as proteins and nucleic acids, but also because of their sensitivity to external stimuli. PEs used for these applications are generally natural PEs such as HA, CS, sodium alginate, characterized by biocompatibility [154,155,156]. Khademhosseini et al. [157] developed a biocompatible support based on PLL and HA allowing cell adhesion and development. The multilayer consisting of the two PEs was deposited on a glass support and used to grow and differentiate two types of cells (stem cells, hepatocytes, and fibroblasts). The obtained results demonstrated the viability of the support for cell culture and stability of about five days. A more recent example is proposed by Zhang et al. [158] that obtained a vascular patch by alternative deposition of heparin and CS on a decellularized substrate coated with polyurethane. In addition to high biocompatibility, the multilayer obtained is also characterized by a good adhesion capacity for endothelial cells, in vivo tests demonstrating implant viability up to five months as compared to only two weeks in the case of the material without CS and heparin.

#### 6.1.3. Thin Films with Antimicrobial Activity

A large number of natural PEs have inhibitory activity on microorganisms, their assembly in multilayers finding applications in the fabrication of non-adhesive or antibacterial films. These systems can be obtained by the LbL deposition of PEs, but also by the formation of multilayers between PEs and inorganic compounds with antibacterial activity, such as silver nanoparticles. For instance, Neto et al. obtained LbL films incorporating silver nanoparticles which demonstrated complete inhibition of *Candida albicans* and *Staphylococcus aureus* [159]. Among the PEs, the most used are CS and HA. Table 2 presents some examples of PEMs as antimicrobial systems.

The increase in the resistance of microorganisms to antibiotics and the prolonged exposure to pathogens makes it necessary to develop innovative systems to prevent microbial contamination and to treat possible infections caused by them. An example of encapsulating an antibiotic in a multilayer is presented by Albright et al. [109]. They fabricated a multilayer based on poly(vinylcaprolactam) and PMA and cross-linked it with ethylenediamine, removing the poly(vinylcaprolactam) from the multilayer. The formed multilayer was used to incorporate two broad-spectrum antibiotics: gentamicin and polymyxin B. The antimicrobial activity of the obtained multilayer was tested on two bacterial strains, *Streptococcus aureus* and *Escherichia coli*, demonstrating not only inhibitory activity on the tested bacterial strains but also a controlled-release of the antibiotic, which appeared as a result of the acidification of the environment under the action of secondary metabolites released by the microorganisms (lactic acid, acetic acid). Ivanova et al. [120] reported the construction of antimicrobial composite materials based on the LbL assembly of HA and aminocellulose (AC) on monobutyl ester of poly (methylvinyl ether/maleic) acid template. The antibiofilm formation activity of the multilayered particles obtained was assessed, demonstrating 94% reduction of *Escherichia coli* and 40% reduction of *Streptococcus aureus* development. Similarly, Francesko et al. [121] assembled thin films by LbL deposition of silver nanoparticles decorated with CH or AC, and HA. The composite materials obtained exhibited inhibitory activity against *Streptococcus aureus* and *Escherichia coli.*

#### 6.1.4. Enzyme-Based Biosensors for Medical Applications

Biosensors have been used as versatile biomedical devices for the identification and quantification of biological signals. Their many advantages, including the high specificity, sensitivity, and ease of use [163,164] raised an intense interest in the scientific world for the development of new and innovative biosensors. Their limitations are related to the low stability and the costs associated with the production of biomolecule-based sensors [164]. Among the materials that are frequently used for the fabrication of biosensors, the most studied ones are conducting polymers [164], gold nanoparticles [165], gold nanorods [166], and carbonaceous materials (multiwalled carbon nanotubes, graphene oxide, mesoporous carbon etc.) [167,168,169,170]. The sensing element can be represented by antibodies [171], DNA [172], or enzymes [173,174,175,176]. The following section will focus on the use of enzymes for the fabrication of biosensors.

The fabrication of biosensors for medical applications based on the immobilization of enzymes in multilayers is a subject intensively studied in recent years, as evidenced by the number of scientific papers in this field. The use of PEMs for the incorporation of active molecules ensures enhanced stability of the biosensor, a selective barrier for the substrate analyzed, and will also prevent the active molecule from bursting out. Among the types of biosensors for medical applications, electrochemical and optical biosensors are the most used ones.

The use of multilayers in the fabrication of electrochemical biosensors is based on the electrical properties of the PEs and their ability to interact with molecules with oppositely charged functional groups, in this case, with proteins and enzymes. Biosensors based on enzymes have specific receptors for certain types of substrates and the ability to mediate a chemical reaction between the receptor and the analyte, allowing the conversion of the chemical response into an electrical response by using an electrode [7]. Additionally, the specificity of the chemical reactions catalyzed by enzymes is one of the most important advantages on enzyme-based biosensors. Table 3 presents some examples of electrochemical biosensors obtained by the immobilization of enzymes into multilayers.

Optical biosensors are based on the interaction of the optical field with a biorecognition element. The detected signal can be generated directly by the interaction of the probe with the transducer (label-free biosensor) or by applying a colorimetric fluorescent or luminescent method (label-based biosensor). Table 4 presents some examples of PEM-based optical biosensors.

### 6.2. Environmental Applications

Over the past few decades, water pollution has become one of the most important problems of our society. As stated by governmental organizations and researchers [184,185,186,187,188,189,190], emerging pollutants are contaminants from various classes of compounds, commonly present in water and wastewater as a result of human activities and which are posing serious risks to human health and ecosystems. A list of pollutants classified as contaminants of high concern is periodically updated by the Norman Network [191], some of the most well-known emerging contaminants (ECs) being dyes, pesticides, endocrine disruptors, pharmaceuticals (diclofenac, naproxen, ibuprofen, antibiotics etc.), and household and personal care products (disinfectants, parabens, fragrances, insect repellents, UV filters). ECs are known to be resistant to degradation, highly persistent in aqueous media, and to potentially have a hazardous effect on ecosystems. Among the most concerning risks associated with the presence of ECs in water, one can mention bioaccumulation, toxicity, carcinogenic potential, and endocrine disruption [188].

**Figure 8:**
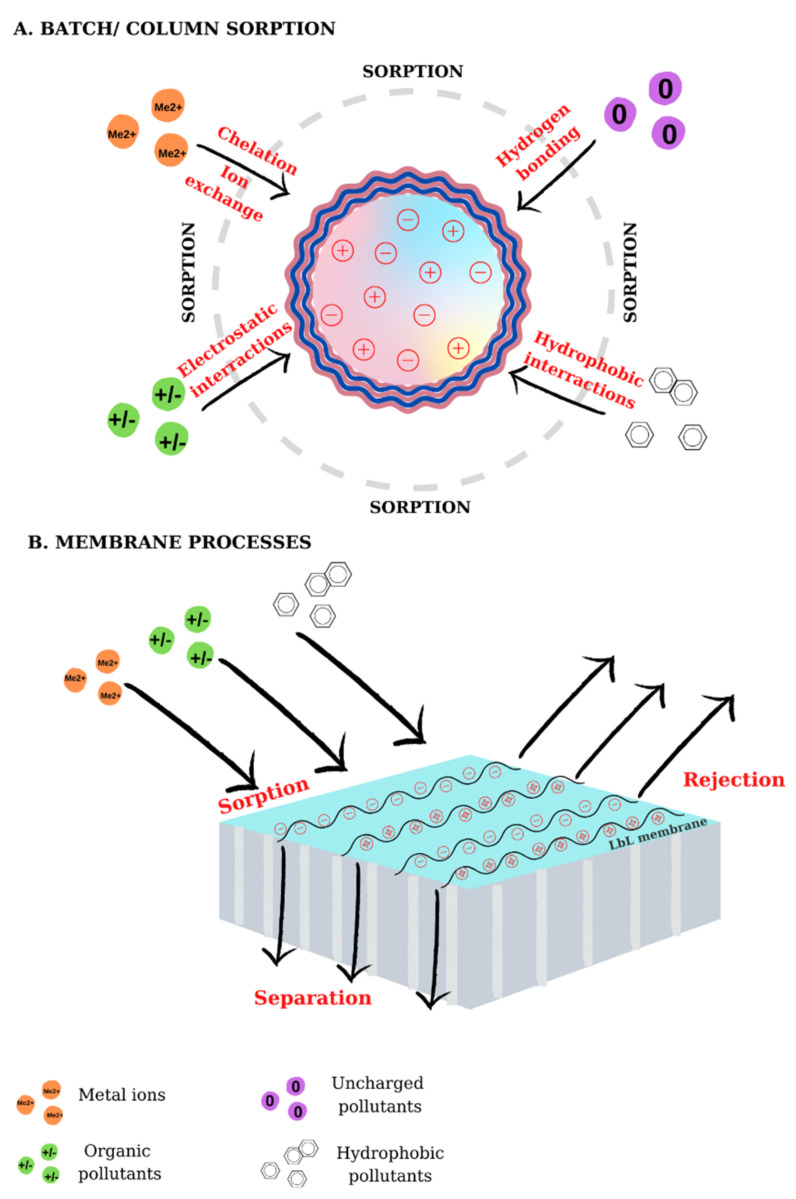
The sorption/rejection of pollutants by LbL-modified colloids (**A**) and membranes (**B**).

The use of conventional technologies for wastewater treatment proved ineffective in the removal of emerging pollutants because of their complexity and low concentration. To diminish this threat, the scientific community focused its attention on the development of new and innovative techniques to retain micro-pollutants from water/wastewater. The use of multilayers for environmental applications is focused in three main directions: the fabrication of composite membranes, desalination membranes, and the synthesis of sorbents, all able to retain/reject pollutants from surface water or wastewaters (Figure 8).

Using the LbL deposition method, the characteristics of membranes or porous materials can be modified, obtaining positively or negatively charged surfaces capable of attracting and repelling chemical species with opposite charges, at the same time facilitating their passing through the membrane pores [192]. Among the PEs used to obtain multilayered membranes are CS [193,194], PAA [194,195], PSS [196,197,198,199], PAH [199,200], and PEI [194,201]. Membrane processes are promising methods for the separation of pollutants. Membranes obtained by layer deposition can be used for the separation of concerning pollutants from water, such as pharmaceuticals, dyes and metal ions using ultrafiltration (UF), reverse osmosis (RO), or nanofiltration (NF), as demonstrated by many authors [202,203,204,205,206,207].

Ilyas et al. [200] used the LbL deposition of PEs for the development of nanofiltration (NF) membranes. The NF membranes based on PAH and PAA prepared at pH = 6/6 had a dense structure which enhanced the retention of the organic pollutants tested (atenolol, sulfamethoxazole, atrazine, naproxen, and bezafibrate) while the membranes prepared at pH = 3.5/3.5 and pH = 6/3.5 had a better retention capacity for charged micropollutants. In another study, the same team studied the modification of an ultrafiltration membrane by PEs deposition to obtain a membrane with a sacrificial layer, which can be easily removed after fouling, by a rinse and backwash method. The PAH/PAA membrane showed good retention capacity for sulfamethoxazole and $S{O_{4}}^{-2}$ ions [202].

Rajesh et al. [201] developed a PEs based membrane obtained by LbL deposition of branched PEI and PAA on a polyacrylonitrile (PAN) nanofibrous material. The obtained membrane showed a high-water flux and 98.7% retention of MgSO_4_. Similarly, de Grooth et al. [103] fabricated a PDADMAC/PSS NF membrane. Their results demonstrated a very high retention capacity for ionic species: 79% retention of CaCl_2_, 71% retention of NaCl, and 71% retention of Na_2_SO_4_. The separation performance of PEs based membranes is influenced by various factors such as the concentration of the PEs solutions used in the deposition, the nature of the last layer deposited, and the pH of the solution to be used for filtration [199]. Baburaj et al. [207] used PEI/PAA and CS/PAA membranes for the separation of two dyes (methylene blue and Coomassie brilliant blue) from water. The two membranes tested showed good removal efficiency for dyes, the amount of pollutant retained being dependent on the type of PE used and the number of deposited bilayers, and also on the pH of the dyes solution.

Abtahi et al. [208] used a PAH/PAA multilayered-UF membrane for the separation of diclofenac, naproxen, ibuprofen, and 4-nonyphenol from simulated wastewater. The membrane obtained by LbL deposition of polyelectrolytes showed very good results in terms of ECs removal (up to 77% diclofenac, 56% naproxen, 44% ibuprofen, and 70% 4-nonyphenol).

The absence of sufficient freshwater resources for drinking in remote areas of the globe is one of the most important challenges our society is facing. To address this problem, intensive research was conducted for the development of new and efficient technologies to use alternative sources of water. Membrane-based technologies (NF, ultrafiltration, and reverse osmosis) are among the most important processes implemented for seawater desalination and PE-based membranes are becoming more and more well-known [209].

As shown by Lajimi et al., the performance of modified NF membranes depends on the number of PE layers deposited and the pore size of the membrane used as a support for the deposition of PEs [210]. The PE deposition approach can also be used in RO applications. Toutianoush et al. deposited PVA and PVS on a (PAN/poly(ethyleneterephtalate) support. The membrane showed complete rejection of NaCl and sulfate ions, and enhanced separation of magnesium, calcium, and sodium ions [211]. Fadhillah et al. [198] demonstrated the feasibility of water purification by UF. The researchers constructed a PAH/PAA multilayer on a polysulfone membrane and tested its ability to reject sodium ions. The results obtained showed a 65% salt rejection for the membrane with 120 PE bilayers and 58% salt rejection for the membrane with 60 bilayers under the tested conditions. In a more recent paper, Suwaileh et al. [212] developed a forward osmosis membrane by dipping a polyethersulfone UF membrane in PDADMAC and sodium carboxymethyl cellulose solutions. The separation performance of the membrane was assessed using solutions of NaCl of different concentrations. The results obtained allowed the authors to determine the optimal operation parameters.

Another application of multilayered materials is the retention of pollutants from water/wastewater using sorbents capable of interacting with pollutants of interest such as heavy metal ions [213,214], or refractory organic compounds [215]. These pollutants are important because of their high stability and their bio-accumulative effect. The sorbents used in their removal can be fabricated by LbL deposition of PEs on charged supports, obtaining composites with good sorption properties. Many sorbents based on PEs have been used in the retention of pollutants from water/wastewater, the most notable systems being those based on weak PEs, which possess ionizable functional groups that can interact with charged pollutants. Some of the most used PEs used in the fabrication of sorbents are PEI, PAA, CS, PAH, etc.

An example is proposed by Bucatariu et al. [87], which tested the retention properties of a multilayer consisting of PEI and PAA, cross-linked with GA after the formation of the silica/(PEI/PAA) composite. The two multilayers obtained using PEI with the linear and branched structure were used in sorption studies of four heavy metal ions (Cu^2+^, Ni^2+^, Co^2+^, Cd^2+^). The results demonstrated the ability of the sorbent to retain the metal ions tested. The same team reported the construction of PE-based sorbents with a high affinity for metal ions [213]. The authors developed composite materials by LbL deposition of PEI, PLL, PVA, and PAH, as polycations and PAA as polyanion on a silica support. The sorption experiments realized using simulated contaminated water and real water showed that the sorption capacity is dependent on the number of functional groups able to interact with metal ions.

PEs-based sorbents can also be used for the removal of refractory organic pollutants from wastewater. Ding et al. [215] prepared micro-shells sorbents by LbL deposition of CS and sodium alginate on a melamine formaldehyde support. The microparticles obtained were subsequently used for the removal of 2,4-dichlorophenol and salicylic acid from aqueous solutions. The materials presented fast kinetics and a good sorption capacity for the organic compounds tested. Bucatariu et al. [88] obtained composite materials with good sorption capacity by depositing PEI/PAA and PVA/PAA multilayers on silica microparticles and cross-linking the polycation chains with GA. The sorbents were used for the removal of two organic dyes (Congo red and bromocresol green) from an aqueous solution in multiple sorption cycles. Between the tested composites, ${Silica/\left(PEI/PAA\right)}_{4.5}$ composites exhibited the highest affinity for the anionic dyes. As highlighted by a series of studies, the sorption depends on the PEs used, the number of layers deposited, the type and degree of cross-linking, but also on the molecular architecture of the compound to be removed from solution [80,216].

## 7. Conclusions

This review presented the main methods of fabrication, properties, and applications of PEMs. From the introduction of LbL deposition method in the 1960s, this technique evolved rapidly, imposing as a versatile, economical, and simple method for surface modification. The properties of the obtained materials can easily be tailored by changing the deposition method and parameters, as highlighted by the research conducted in the field of LbL deposition of PEs. Due to the low manufacturing costs and easy chemistry modification, PE multilayered surfaces are very popular and frequently used in the medical field and wastewater treatment. Therefore, LbL PE surfaces, decorated with a high and diverse number of functional groups, are characterized by a high selectivity towards different biologically active compounds or pollutants. In this way, many studies have been focused on the enhancement of loading/release or sorption/separation performances of these types of LbL modified surfaces. The formation of new LbL architectures with high permeability and selectivity, rejection and sorption capacities, and antifouling properties are the biggest challenges for PE researchers. Using “soft” PEs, deposited onto “hard” inorganic surfaces could result in an “upgraded” material with synergic properties in capture, transport, and subsequent release of some targeted compounds. The tunable properties and the facile deposition methods make PEMs an ideal material for a long list of applications. Tissue engineering applications, controlled-release systems, sorbents for pollutants removal, food packaging, and biosensors can be obtained based on PEMs. Nevertheless, the extensive research efforts made in the field of PEMs is far from being sufficient. Subsequent work is needed to fully understand the mechanism of LbL deposition of PE and to develop new and innovative applications.

## Figures and Tables

**Table 1:** Active compounds incorporated in multilayers.

Active Compound	Therapeutic Class	Incorporation Method	Polyelectrolytes Used	Reference
Diclofenac, Indomethacin	Analgesic	PE-AC interaction	PEI–PAA	[97]
Ibuprofen	Analgesic	PE-AC interaction	CS–dextran sulphate CS–carboxymetylcelullose	[127]
Rifampicin	Antibiotic	Incorporation into hollow capsules	PVP–PMA	[128]
Tetracycline	Antibiotic	PE-AC interaction	PAA–poly(L-lysine)	[129]
Gentamicin	Antibiotic	PE-AC interaction	PSS–PAH	[130]
Benzydamine	Anti-inflammatory agent	PE-AC interaction	CS–casein–poly(lactic acid)	[131]
Artemisin	Chemotherapeutic agent	PE-AC interaction	CS–gelatin–ALG	[132]
Paclitaxel Fluorouracil	Chemotherapeutic agent	PE-AC interaction	CS–dextran sulphate	[133]
Mitoxantrone	Chemotherapeutic agent	Incorporation into hollow capsules and PE-AC interaction	PLL–PEG–block-poly(L-aspartic acid)–liposomal nanoparticles	[134]
Doxorubicin	Chemotherapeutic agent	Incorporation into hollow capsules	CS–HA	[135]
Insulin	Hormone	PE-AC interaction	Poly(malic acid)–CS	[136]

**Table 2:** Multilayers based on polyelectrolytes with antimicrobial activity.

Microorganism	Polyelectrolytes Used	Reference
*Escherichia coli*	CS–heparin–PET	[160]
	CS–lignosulphonate	[161]
	CS–HA	[159]
*Staphylococcus* *aureus*	CS–alginate	[159]
	CS–HA	[159]
	PEI–PAA	[162]
*Candida albicans*	CS–alginate	[159]
	CS–HA	[159]
*Pseudomonas fluorescens*	PEI–PAA	[162]

**Table 3:** Examples of electrochemical biosensors based on the immobilization of enzymes into multilayers.

Active Molecule	Polyelectrolytes Used	Substrate Analyzed	Reference
Phytase	PAH	Phytic acid	[173]
Glucose oxidase	CS	Glucose	[174]
Monoamine oxidase B	PEI	Dopamine	[124]
Horseradish peroxidase	PAH	Hydrogen peroxide	[175]
Urease	Polyaniline/CS/carboxymethylpullulan	Urea	[176]
Uricase	PEI PDADMAC	Uric acid	[177]
β-glucanase Glucose oxidase	CS	β-glucan	[178]
Choline oxidase	PEI PDADMAC	Choline	[179]

**Table 4:** Examples of optical biosensors based on the immobilization of enzymes into multilayers.

Active Molecule	Polyelectrolytes Used	Substrate Analyzed	Reference
Urease	PAH/Cresol Red-PSS	Urea	[180]
Tyrosinase	PDADMAC	L-DOPA	[181]
Glucose oxidase	CS PSS	Glucose	[123]
Organophosphorus hydrolase	CS/poly(thiophene-3-acetic acid)	organophosphorus compounds	[182]
Urease Arginase	PDADMAC/PSS	L-arginine	[183]

## Data Availability

The data presented in this study are available on reasonable request from the corresponding author.
